# Evaluating Microlearning for Faculty Development in Medical Education: Mixed Methods Pilot Study

**DOI:** 10.2196/87980

**Published:** 2026-03-11

**Authors:** Darci L Lammers, Jeffrey B Geske, Jane A Linderbaum, Michael W Cullen

**Affiliations:** 1 Cardiovascular Education Mayo Clinic Rochester, MN United States; 2 Cardiovascular Diseases Mayo Clinic Rochester, MN United States

**Keywords:** faculty development, medical education, continuing medical education, knowledge transfer, behavioral change, instructional design, digital learning, scalability, sustainability, cardiovascular diseases, mixed methods, knowledge retention, asynchronous learning, professional development

## Abstract

This mixed methods pilot study evaluates the feasibility and effectiveness of microlearning for faculty development in cardiovascular education. Microlearning appears feasible and well-received for faculty development, offering a scalable, flexible approach.

## Introduction

Traditional faculty development relies on time-intensive, in-person sessions, limiting participation for clinician-educators balancing clinical and administrative duties [1,2].

Microlearning delivers brief, focused segments (≤15 min) aligned with objectives [3,4]. Grounded in cognitive science, it reduces cognitive load and enhances retention by presenting information in small units [5]. For busy clinician-educators, microlearning offers flexible, asynchronous learning that addresses time constraints and supports targeted, on-demand modules for skill application [3,4,6], and it improves engagement and aligns with digital learning preferences [6,7].

However, research has focused only on the short-term outcomes of microlearning, leaving gaps in sustained knowledge transfer and behavioral change [3,4,6]. Without accessible and time-efficient faculty development approaches, clinician-educators may continue to rely on informal or inconsistent training, potentially compromising the quality of educational assessments, learner outcomes, and the validity of continuing medical education (CME) activities.

We selected CME multiple‑choice question (MCQ) development as our intervention topic based on an internal needs analysis indicating that faculty responsible for creating CME assessment items receive little or no structured guidance in item writing. This task was identified locally as a high‑frequency responsibility in which faculty desired more support. MCQ development serves as a practical context to evaluate microlearning for clinician‑educators.

This mixed methods pilot evaluates microlearning feasibility and effectiveness in cardiovascular faculty development by assessing learning transfer, satisfaction, and 4‑month knowledge application.

## Methods

We conducted a sequential explanatory study guided by Kirkpatrick’s framework [8] to assess a microlearning module for faculty development in cardiovascular education. The Mayo Clinic Institutional Review Board reviewed the study and determined it to be exempt. Participants provided informed consent and could withdraw any time. Those completing all components received US $200 remuneration. All data were deidentified. Quantitative analysis was prioritized, with qualitative interviews used to explain and contextualize test results.

Cardiology clinician‑educators responsible for CME MCQs were recruited via email. Eligibility required current responsibility for board‑style MCQ development and no formal training or related coursework within 6 months. Of the 75 identified faculty, 34 met the criteria; 8 enrolled and completed all the components.

The pretest (18 items across 4 sections; total 400 points, scored in the learning management system) preceded each corresponding microlearning segment (Multimedia Appendix 1). The module comprised 4 short videos, a quick‑reference guide, and an MCQ‑writing template, delivered asynchronously via the cardiology CME learning management system for 3 months. After a 15‑item satisfaction survey (Multimedia Appendix 2), access was discontinued. An identical posttest occurred 4 months after completion to assess retention; 2 weeks later, semistructured Teams interviews explored application and perceptions (Multimedia Appendix 3).

We compared pre/post scores using the 2‑sided Wilcoxon signed-rank test (reporting Pratt method including ties), calculated matched-pairs rank-biserial effect sizes, and summarized distributions with medians (IQRs).

Survey responses were summarized descriptively and used to inform preliminary qualitative themes. We conducted a structured hybrid deductive–inductive analysis. The lead investigator completed line‑by‑line coding by using an a priori codebook derived from the interview guide, microlearning constructs, and Kirkpatrick’s model, with inductive codes added as needed. An education researcher not involved in the study independently reviewed all the coded transcripts. Discrepancies were resolved through consensus, and the codebook was refined iteratively.

Quantitative and qualitative data were integrated by the lead and co-lead investigators, who jointly analyzed test scores, survey responses, and interview transcripts. Quantitative results informed qualitative coding, enabling exploration of trends and outliers. Both strands were interpreted together to provide a comprehensive understanding of the module’s impact.

## Results

Among the 8 completers, the median pretest score was 366.07 (IQR 338.93-389.28), and the median posttest score was 400.00 (IQR 361.96-400.00). Of the 8 paired scores, 5 improved, 2 were unchanged (ties), and 1 decreased; thus, informative pairs were n=6.

A Wilcoxon signed-rank test (excluding ties) yielded W=5.0, *P*=.25; using the Pratt method (including ties), W=7.0, *P*=.18. The matched-pairs rank-biserial correlation was 0.52, indicating a moderate positive effect of the intervention.

Satisfaction survey responses showed strong agreement across all dimensions, with participants endorsing the module’s relevance, clarity, and flexibility (Table 1).

**Table 1:** Quantitative summary of the satisfaction survey responses (N=8).

Survey item	Strongly agree, n (%)	Agree, n (%)
Videos were engaging	6 (75)	2 (25)
Video length was appropriate	8 (100)	0 (0)
Modular format was effective	7 (88)	1 (12)
Prepared to write board-style review questions	6 (75)	2 (25)
Quick reference guides were valuable	8 (100)	0 (0)
Pretest helped gauge prior knowledge	5 (63)	3 (37)
Pretest guided learning	5 (63)	3 (37)
Posttest reinforced learning	5 (63)	3 (37)

Qualitative analysis revealed three main themes: (1) appreciation for the concise, flexible format; (2) direct application of learned principles in educational practice; and (3) high perceived value and satisfaction (Table 2). Time constraints remained a barrier to engaging in faculty development, but microlearning was widely endorsed as effective and scalable.

**Table 2:** Key themes from qualitative interviews.

Theme	Subthemes	Representative findings
Value of microlearning format	Flexibility and time efficiency Reduced cognitive load Point-of-need learning	Microlearning was praised for fitting into busy schedules and enabling learning in short, focused bursts
Knowledge application and transfer	Direct application to CME^a^ Immediate use of quick-reference codes and template Knowledge shared with colleagues	Learned principles were immediately used in CME MCQ^b^ development and shared with colleagues. Modules will serve as an ongoing reference
Perceived value and satisfaction	High ratings (excellent/very good) Clear, concise, relevant content Multimedia quality	Participants appreciated the clarity, relevance, and production quality of the modules and resources
Barriers to faculty development	Time constraints Administrative duties Lack of protected time from clinical responsibilities	Common barriers included competing clinical/administrative demands and lack of dedicated time for learning
Suggestions for improvement	Familiar platforms Time estimates for modules Follow-up opportunities Resource access	Recommendations included hosting on familiar organizational platforms, adding time estimates, and offering structured follow-up with an expert

^a^CME: continuing medical education.

^b^MCQ: multiple choice question.

## Discussion

We demonstrated that a microlearning intervention for cardiovascular faculty development was feasible, well-received, and associated with improved knowledge scores and perceived application of skills at 4 months, aligning with the objectives to assess learning transfer, satisfaction, and sustained knowledge application.

Findings align with prior evidence that microlearning enhances engagement, skill acquisition, and retention in health education [1,3]. The module’s brevity, relevance, and asynchronous access likely contributed to its effectiveness. The 4-month follow-up addresses a gap in prior faculty-focused studies, which often lack longer-term data [2]. Microlearning can improve learning outcomes and self-efficacy, further supporting its value in CME [9].

By integrating quantitative data from pretests and posttests and satisfaction surveys with qualitative insights from interviews, investigators contextualized statistical findings with participant perspectives. Quantitative results informed preliminary qualitative coding, enabling exploration of trends and outliers. This approach provided a more comprehensive understanding of the module’s impact and feasibility, revealing factors and barriers not apparent from a single data type.

Mixed methods revealed both measurable gains and contextual insights, including barriers such as time constraints. Participant recommendations such as hosting modules on familiar platforms and providing time estimates for each section may further improve engagement.

Limitations include the single-institution setting, small sample size, exclusion of tied pairs in the primary analysis, lack of a control group, and potential response bias. The 4-month follow-up may not capture durable behavior change. Integrating quantitative and qualitative methods enhanced understanding but may introduce interpretive complexity.

Overall, microlearning appears to be a scalable, flexible approach to faculty development, well-suited to clinical educators. Institutions should consider implementing microlearning modules with structured follow-up to reinforce learning. Future research should use larger, more diverse samples, include control groups, and extend follow-up to evaluate long-term behavioral and organizational outcomes.

## Data Availability

The datasets generated and analyzed during this study are not publicly available because participant consent for data sharing was not obtained. Data may be available from the corresponding author upon reasonable request and with appropriate institutional approvals, provided such sharing complies with participant privacy and ethical guidelines.

## Appendix

Multimedia Appendix 1 Microlearning pretest and posttest.

Multimedia Appendix 2 Post course evaluation.

Multimedia Appendix 3 Follow-up interview guide.

## Abbreviations

CME: continuing medical education
MCQ: multiple choice question
