# Supplementary material for: Evaluating Microlearning for Faculty Development in Medical Education: Mixed Methods Pilot Study
Source: JMIR Med Educ. 2026 Mar 11;12:e87980. doi: 10.2196/87980 (PMC13019028; doi:10.2196/87980)
Supplement: Multimedia Appendix 1 [file mededu_v12i1e87980_app1.docx]

**Authoring CME Questions Pre/Posttest**

**101**

**What is a crucial aspect when authoring multiple-choice questions for CME in terms of alignment with content standards?**

Options:

A) Ensuring that your questions replicate current board review questions

B) Matching the standard, such as the ABIM Blueprint

C) Including distractors that are different in length to challenge learners

D) Including questions that test basic skills and concepts

**In what way do well-designed multiple-choice questions contribute to facilitating life-long learning among medical providers?**

Options:

A) By encouraging learners to take other CME courses

B) By Including distractors that are different in length to challenge learners

C) By reinforcing core content and identifying knowledge gaps

D) By Including questions that test basic skills and concepts

**102**

**Asking a knowledgeable colleague to review your questions and having individuals unfamiliar with the subject matter attempt to answer them is important to the construction of good multiple-choice questions. Why is this peer review process valuable in question development?**

Options:

A) It includes the opinion of one other person, creating a well-rounded question.

B) Helps identify irrelevant levels of difficulty in questions.

C) Having fellows answer questions will tell you whether they’re ready to practice medicine.

D) The process ensures that only the toughest questions are included in assessments.

**In what way does providing supplemental resources for each multiple-choice question benefit learners?**

Options:

A) It simplifies the assessment process

B) It encourages independent learning and exploration

C) It reduces critical thinking demands

D) It limits access to external information

**Why is it important for authors designing scenario-based multiple-choice questions to ensure alignment with real-world applications?**

Options:

A) To challenge learners by presenting only complex scenarios

B) To challenge learners' ability to apply knowledge beyond theoretical contexts

C) To limit responses within theoretical boundaries only

D) To encourage learners to compare and contrast different clinical options

**In developing multiple-choice questions for CME, what is the significance of aligning questions with clear learning objectives?**

Options:

A) It makes questions more ambiguous, challenging learners further.

B) It allows for testing multiple learning objectives within a single question.

C) It ensures that each question assesses a specific piece of knowledge.

D) It limits the scope of questions, making them more challenging.

**What should educators watch for when developing MCQs to ensure that learners can answer based solely on the provided information without external cues?**

Options:

A) Including typos to test attention to detail.

B) Providing irrelevant levels of detail to challenge learners.

C) Avoiding cues that benefit savvy test-takers but do not assess knowledge.

D) Incorporating controversial topics to encourage additional research.

**What is a key consideration when identifying distractors for multiple-choice questions?**

Options:

A) Making them longer than the correct answer for complexity

B) Ensuring they are similar in format to the correct answer

C) Including distractors from previous lesson content

D) Using distractors that are factually accurate but irrelevant

**What is a key aspect when developing lead lines for multiple-choice questions?**

Options:

A) Making them lengthy to provide more context

B) Pairing them with incorrect answers for variety

C) Ensuring they directly pose the specific question

D) Including references within each lead line

**103**

**What is the primary reason for avoiding True/False and Yes/No questions in assessments?**

Options:

A) They encourage guessing

B) You shouldn’t avoid them—they’re fine

C) They simplify the evaluation process

D) They encourage learner debate

**What is the primary drawback of including catch-all options like "all of the above" or “none of the above” in multiple-choice questions?**

Options:

A) They increase learner engagement

B) They discourage guessing behavior

C) They hinder accurate assessment of knowledge

D) They simplify the decision-making process for learners

**How does using negative phrasing in a question impact learner performance?**

Options:

A) It enhances comprehension and retention

B) It simplifies decision-making for learners

C) It makes questions difficult to understand

D) It encourages creative problem-solving skills

**Identifying the testing point before crafting a question stem ensures relevance and eliminates unnecessary information. How does this approach contribute to effective question development?**

Options:

A) It helps in creating longer questions that challenge the test-takers.

B) It ensures that the question stem is concise and directly related to the intended question.

C) It leads to including irrelevant details that may confuse the learners.

D) It encourages the use of complex language to enhance difficulty.

**Why should distractors be carefully crafted to be plausible yet clearly distinguishable from the correct answer?**

Options:

A) To confuse learners and increase difficulty

B) To ensure that guessing is discouraged

C) To promote deep critical thinking and analysis

D) To provide hints towards the correct answer

**104**

**Review the board style question below and identify the problem with its construction.**

| Learning Objective: Interpret pacemaker electrocardiography and apply findings to patient management.  Question Stem: A 66-year-old man had a VVI pacemaker placed for syncope in the setting of sick sinus syndrome. Since the pacemaker placement, he has felt weak and lightheaded with pulsations in his neck. He is currently living at home with his wife and one small dog whom he takes frequent walks with. His 12-lead ECG is shown.  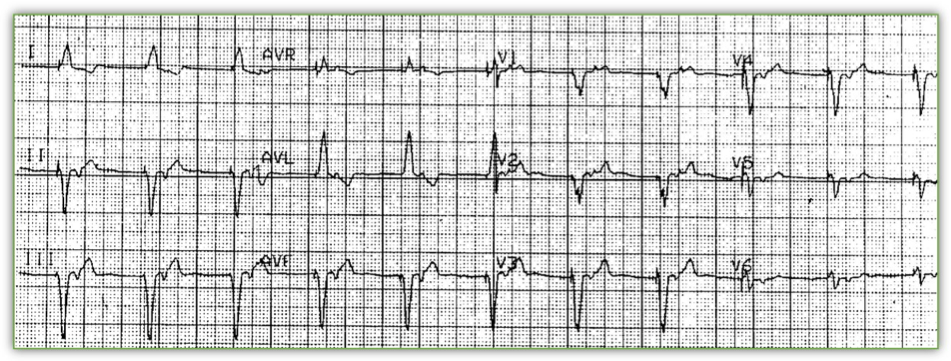  Lead Line: What is the best approach for management?   1. Upgrade to a DDD pacemaker 2. Switch to VOD mode at 60 bpm 3. Readjust the lead voltage output 4. Upgrade to a CRT device   Correct answer: A  Rationale: This patient has "pacemaker syndrome" from retrograde conduction to his atrium during  VVI pacing causing his symptoms and hypotension. The optimal approach is to upgrade to a DDD pacemaker to allow antegrade AV conduction.  References: Epstein AE, DiMarco JP, Ellenbogen KA, et al. 2012 ACCF/AHA/HRS focused update incorporated into the ACCF/AHA/HRS 2008 guidelines for device-based therapy of cardiac rhythm abnormalities: a report of the American College of Cardiology Foundation/American Heart Association Task Force on Practice Guidelines and the Heart Rhythm Society. J Am Coll Cardiol. 2013 Jan 22;61(3):e6-75. doi: 10.1016/j.jacc.2012.11.007. Epub 2012 Dec 19. PMID: 23265327 |
| --- |

Options:

1. Negative phrasing was used
2. Irrelevant information is included in the question stem
3. The response options provide clues to the correct answer
4. There is more than one correct answer

**Review the board style question below and identify the problem with its construction.**

| Learning Objective: Identify risk factors for sudden cardiac death in adults with repaired tetralogy of Fallot.  Question Stem: A 51-year-old man with tetralogy of Fallot status post Blalock-Taussig shunt at age 2, transannular patch repair (age 6), surgical pulmonary valve replacement (PVR) (age 38), and transcatheter PVR at age 45, is admitted with NYHA Class III limitations and pulmonary edema requiring diuresis. He is treated with diuretics and investigations are undertaken.  Lead Line: Which of the following findings from his investigations is associated with increased risk of sudden cardiac death?   1. QRS duration > 180msecs 2. LV end-diastolic pressure > 16mmHg 3. NYHA Class II or higher 4. All of the above   Correct answer: D  Rationale:  The incidence of sudden cardiac death (SCD) in adults with repaired tetralogy of Fallot ranges between 1.2% to 3.0% per decade. Implantable cardiac defibrillators (ICDs) are indicated in adults with congenital heart disease (CHD) resuscitated from SCD and in those with spontaneous sustained ventricular tachycardia (VT) after a thorough workup has excluded any secondary causes.   Identifying which adults with tetralogy of Fallot are most likely to benefit from primary prevention ICD is challenging. Factors associated with SCD in patients with TOF include non-sustained ventricular tachycardia, elevated pulmonary capillary wedge pressure, reduced LV ejection fraction, and prolonged QRS duration > 180msecs. In one of the largest studies to date, 465 adults with repaired tetralogy of Fallot were followed for a median of 13.6 ± 8.2 years. The following were independent risk factors for death and/or transplant: age >42 years, atrial fibrillation, ≥moderate QRS fragmentation, left ventricular ejection fraction <50%, right ventricular end-diastolic pressure >16 mm Hg, and left ventricle end-diastolic pressure >16 mm Hg.   Current guidelines recommend primary prevention ICD in adult tetralogy of Fallot patients who meet standard criteria backed by clinical trial evidence - that is a 2-ventricle circulation with a systemic left ventricle ejection fraction ≤ 35% and NYHA Class II-III symptoms. Based on the current guidelines the patient should be considered for primary prevention ICD.  References:  Khairy P, Van Hare GF, Balaji S, Berul CI, Cecchin F, Cohen MI, Daniels CJ, Deal BJ, Dearani JA, Groot Nd, Dubin AM, Harris L, Janousek J, Kanter RJ, Karpawich PP, Perry JC, Seslar SP, Shah MJ, Silka MJ, Triedman JK, Walsh EP, Warnes CA. PACES/HRS expert consensus statement on the recognition and management of arrhythmias in adult congenital heart disease: developed in partnership between the Pediatric and Congenital Electrophysiology Society (PACES) and the Heart Rhythm Society (HRS). Endorsed by the governing bodies of PACES, HRS, the American College of Cardiology (ACC), the American Heart Association (AHA), the European Heart Rhythm Association (EHRA), the Canadian Heart Rhythm Society (CHRS), and the International Society for Adult Congenital Heart Disease (ISACHD). Can J Cardiol. 2014 Oct;30(10):e1-e63.   Egbe AC, Kothapalli S, Borlaug BA, Ammash NM, Najam M, Bajwa N, Tarek K, Mathew J, Connolly HM. Mechanism and Risk Factors for Death in Adults With Tetralogy of Fallot. Am J Cardiol. 2019 Sep 1;124(5):803-807. |
| --- |

Options:

1. Negative phrasing was used
2. Answer and distractors are not similar length
3. The author used a “catch all” answer
4. The author included clues in the response options

**Review the board style question below and identify the problem with its construction.**

| Learning Objective: Select appropriate management for patients with mechanical complications of myocardial infarction  Question Stem: A 52-year-old man underwent thromboIytic therapy for an inferior STEMI. Two days later he suddenly develops severe SOB and hypotension and is found to be in acute pulmonary edema. On exam, he is sitting bolt upright in acute respiratory distress with rales in the lung fields but no murmur is audible. BP is 80/50 mmHg and heart rate 110 bpm. A TTE shows hyperdynamic LV function without pericardial effusion but is of poor quality.  Lead Line: What is the next best step in management?   1. Proceed directly to surgery 2. TEE with surgical standby 3. Right and left heart catheterization with saturations 4. Place SG catheter with nitroprusside and dobutamine   Correct answer: B  Rationale: Patients with acute onset of pulmonary edema and hypotension after MI should be evaluated for mechanical complications of MI. Acute papillary muscle rupture is seen with an inferior MI due to the single blood supply to the postero-medial papillary muscle. This is frequently missed by TTE but should be suspected on the basis of the clinical scenario and hyperdynamic LV function. A TEE is required for diagnosis and then urgent surgery is indicated. |
| --- |

Options:

1. Negative phrasing was used
2. Answer contains “correct answer clues”
3. The author used a “catch all” answer
4. Provide at least one supplemental resource

**Dr. Jensen, a world-renowned Physician in Cardiology, just completed writing 10 board style review questions and has thoroughly checked them over for accuracy. What should their next step be?**

Options:

1. Ask a knowledgeable colleague to review the questions and provide feedback
2. Submit the questions to the appropriate personnel for implementation
3. Send an e-mail to their administrative assistant asking about next steps
4. Check the questions over one last time before submission
