# Supplementary material for: Evaluating Microlearning for Faculty Development in Medical Education: Mixed Methods Pilot Study
Source: JMIR Med Educ. 2026 Mar 11;12:e87980. doi: 10.2196/87980 (PMC13019028; doi:10.2196/87980)
Supplement: Multimedia Appendix 2 [file mededu_v12i1e87980_app2.docx]

**Course Evaluation**

**Authoring CME Questions**

Instructions: Please take a few minutes to complete this evaluation of the microlearning course, Authoring CME Questions. You will be asked to respond with your thoughts regarding the videos, quick reference guides, and quizzes.

**Name:___________________________________________________________________________________________**

**Identify your profession:**

- Physician
- Nurse Practitioner
- Physician Assistant
- Other

**Overall, how would you rate this activity?**

- Excellent
- Very Good
- Good
- Fair
- Poor

**The content of this activity prepared me to write board-style questions:**

- Yes
- No

If no, please explain:_______________________________________________________________________________

**This microlearning experience is a comparable substitute for an in-person class:**

- Yes
- No

If no, please explain:_______________________________________________________________________________

**This microlearning experience provided a convenient way for me to receive the education I need without disrupting my clinical responsibilities:**

- Yes
- No

If no, please explain:_______________________________________________________________________________

**Indicate your agreement with the following statements regarding the microlearning content:**

|  | **Strongly Agree** | **Agree** | **Neutral** | **Disagree** | **Strongly Disagree** |
| --- | --- | --- | --- | --- | --- |
| ***The videos were engaging.*** |  |  |  |  |  |
| ***The video length was appropriate for the content.*** |  |  |  |  |  |
| ***Breaking video content in smaller “chunks” enhanced my learning experience.*** |  |  |  |  |  |
| ***After watching the videos, I now feel adequately prepared to write board-style questions.*** |  |  |  |  |  |
| ***The quick reference guides will be a valuable resource for me when I begin writing board style review questions.*** |  |  |  |  |  |
| ***The pretest helped me understand my knowledge level prior to reviewing the microlearning videos and quick reference guides.*** |  |  |  |  |  |
| ***The pretest prepared me for what I needed to learn from the microlearning materials.*** |  |  |  |  |  |
| ***The posttest reinforced my learning of the microlearning videos and quick reference guides.*** |  |  |  |  |  |

**How can we improve the format of this activity?**

***Comments:­­­­­­­­­­­­­­­­­­­______________________________________________________________________________________***

**I would recommend this activity to others:**

- Yes
- No

**If no, please explain:**

**Provide additional comments related to this activity:**
