# Supplementary material for: Evaluating Microlearning for Faculty Development in Medical Education: Mixed Methods Pilot Study
Source: JMIR Med Educ. 2026 Mar 11;12:e87980. doi: 10.2196/87980 (PMC13019028; doi:10.2196/87980)
Supplement: Multimedia Appendix 3 [file mededu_v12i1e87980_app3.docx]

**Course Evaluation**

**Authoring CME Questions**

**Interviewer Guide**

***Interviewee Name:______________________________________________________________________________***

**Profession:**

- Physician
- Nurse Practitioner
- Physician Assistant
- Advanced Practice Nurse
- Other

**Now that three months have passed, how would you rate this activity?**

- Excellent
- Very Good
- Good
- Fair
- Poor

**How did this activity adequately prepare you to write board-style questions?**

Comments:______________________________________________________________________________________

**Did you experience any disruptions when taking this course (e.g., frequent interruptions, clinical emergencies, lack of a suitable learning location.)?**

Comments:______________________________________________________________________________________

**This course was designed using the concept of microlearning. Microlearning is educational content that learners can consume very quickly, often in as little as 90 seconds, and can be presented via any digital modality including text, video, audio (e.g., short snippets of speech), infographics, or images. Would you take another microlearning course on a different faculty development topic? Why or why not?**

Comments:______________________________________________________________________________________

**We recognize that finding time to complete faculty development activities can be a challenge. Would microlearning modules like this help overcome these time barriers to faculty development in the future?**

Comments:______________________________________________________________________________________

**Compared to traditional in-person courses, did you find this experience convenience and easy to incorporate into your daily time management? Why or why not?**

Comments:______________________________________________________________________________________

**How can we improve this educational activity?**

Comments:______________________________________________________________________________________

**Provide additional comments related to this activity:**

**Would you recommend this activity to others:**

- Yes
- No

**Comments:_______________________________________________________________________________________**
